# Liver Abscess after Common Hepatic Artery Embolization for Delayed Hemorrhage Following Pancreaticoduodenectomy: A Case Report

**DOI:** 10.1155/2010/280430

**Published:** 2010-06-13

**Authors:** Yuichi Sanada, Hiroki Kondo, Satoshi Goshima, Masayuki Kanematsu, Yoshihiro Tanaka, Yasuharu Tokuyama, Shinji Osada, Kazuhiro Yoshida

**Affiliations:** ^1^Department of Surgical Oncology, Gifu Graduate School of Medicine, 1-1 Yanagido, Gifu 501-1194, Japan; ^2^Department of Radiology, Gifu Graduate School of Medicine, 1-1 Yanagido, Gifu 501-1194, Japan

## Abstract

A 55-year-old man underwent pancreaticoduodenectomy for bile duct carcinoma in March 2009. The patient developed anastomotic leakage and had a short episode of hemorrhage from the drainage tubes with spontaneous disappearance. CT and upper endoscopy did not reveal the source of bleeding. A massive life-threatening hemorrhage occurred on the 18th postsurgical day. Emergency angiography showed a 2.7-cm pseudoaneurysm of the gastroduodenal artery stump, and hepatic artery embolization was performed. After embolization, an abscess appeared in segments 2/3 of the liver without involving the right lobe. We treated conservatively by drainage and antibiotics. During the course of therapy after embolization, the patient experienced several episodes of high fever but did not develop hepatic failure. On the 68th day after embolization, the abscess had penetrated to the lesser sac, which was immediately treated by percutaneous drainage. Anastomotic leakage was treated by continuous irrigation from the drain, for which complete resolution was achieved by the 34th day after embolization. The patient was discharged 101 days after embolization. Imaging and the clinical course demonstrate a unique mechanism of abscess formation after embolization.

## 1. Introduction

Delayed arterial hemorrhage after pancreaticoduodenectomy (PD) is an uncommon complication (incidence 2–4%) but carries a high mortality, with up to a third of patients dying as a consequence [1]. Early diagnosis and treatment of bleeding are major concerns for pancreatic surgeons. Urgent surgery to control bleeding after PD is difficult, due to tissue scaring and necrosis around the pancreatic anastomosis [2]. 

 Instead, selective angiography and TAE is now considered the standard therapeutic management. This enables the precise localization of a ruptured pseudoaneurysm and allows selective microcoil embolization, providing definitive hemostasis in 63% to 80% of cases [3, 4]. 

An important sign for postpancreatectomy hemorrhage (PPH) is sentinel bleeding [5]. A study of a large number of cases revealed that sentinel bleeding prior to PPH is associated with a mortality of >50%. Therefore, the observation of sentinel bleeding should lead to emergency diagnosis and detection of the source of bleeding to prevent massive blood loss [3]. 

Here we report a case of delayed PPH arising from pseudoaneurysm of the gastroduodenal artery stump. In the present case, because enhanced computed tomography and upper endoscopy could not detect the source of bleeding after becoming aware of the sentinel bleeding, we could not prevent a life-threatening hemorrhage. 

In addition, another important point in the present case is the occurrence of a liver abscess after coil embolization of the hepatic artery. Hepatic infarction and abscess after embolization have been reported in some case series [6]. However, the appropriate therapeutic strategy and management for this complication is still unclear. In the present case, a hepatic abscess in segments 2/3 of the liver occurred after embolization. The morphological changes of the hepatic abscess visualized in CT and detailed observation of the clinical course after embolization provide a mechanism for the formation and disappearance of hepatic abscesses that has not been described previously.

## 2. Case Report

**Figure 1:**
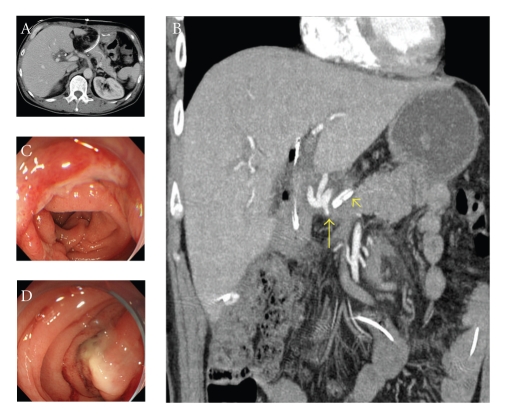
Imaging after sentinel bleeding. (a) CT scan on the 16th day postsurgery shows no abscess or hematoma around the pancreatic anastomosis. (b) The stump of the GDA is 6.0 mm in diameter (arrow), and very close to the drain placed at the anterior aspect of the pancreaticojejunostomy (arrowhead). (c) and (d) an upper endoscopy on the 17th day postsurgery shows ulcerative changes without massive hemorrhage in the gastrojejunostomy (c) and the pancreaticojejunostomy (d).

**Figure 2:**
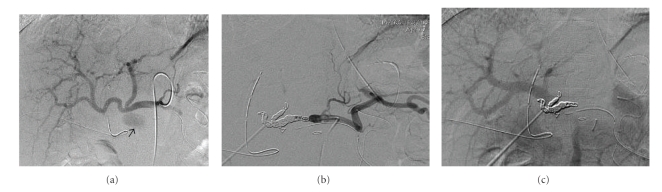
Emergency angiography on the 18th day postsurgery. (a) A 2.7 cm pseudoaneurysm of the GDA stump is observed (arrow). (b) Coil embolization of the hepatic artery, between the proximal and distal side of the pseudoaneurysm was performed. (c) A complete hemostasis was achieved. Obstruction of the portal branches was not observed.

**Figure 3:**
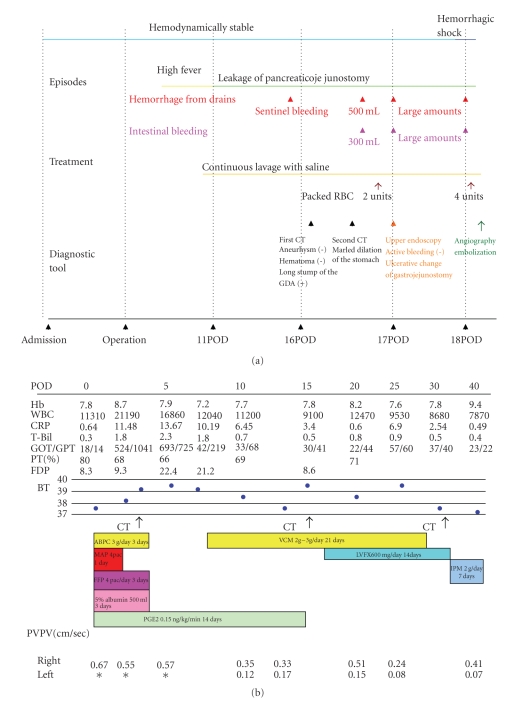
(a) Clinical course during the periods between admission and emergency angiography. (b) The change in laboratory values and treatment after embolization. Hb: hemoglobin (g/dl); WBC: white blood count (per mm^3^); CRP: C-reactive protein; T-Bil: Total bilirubin; GOT: glutamic oxaloacetic transaminase; GPT: glutamic pyrubic transamynase; PT: prothrombin time; FDP: fibrin degradation producrt; BT: body temperature; ABPC: ampicillin; VCM: vancomycin; MAP: packed RBC; FFP: fresh frozen plasma; LVFX: quinolone; PGE2: prostaglandin E2; IPM: imipenem; PVPV: portal vein peak velocity.

**Figure 4:**
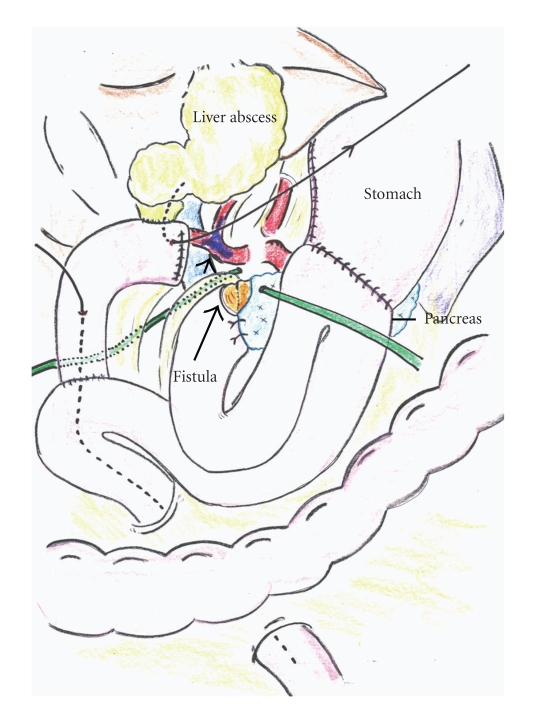
Schematic presentation of the reconstruction and liver abscess after embolization. Drainage tubes, a decompression tube placed in the hepaticojejunostomy, and pancreatic anastomosis are shown.

**Figure 5:**
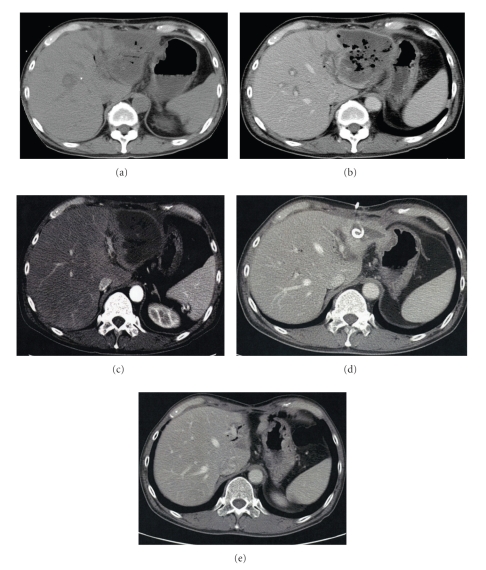
Representative images of CT performed after embolization. (a) 3 days after embolization. (b) 16th day after embolization. (c) 30th day after embolization. (d) 68th day after embolization. (e) 161 st day after embolization.

**Figure 6:**
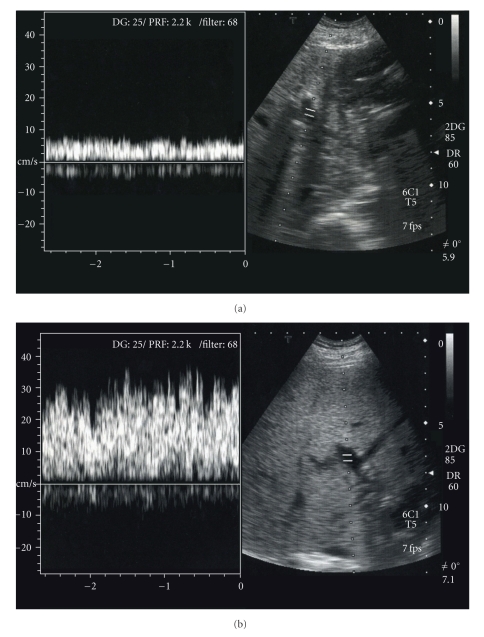
Doppler ultrasonography shows gradual decrease of the portal vein peak velocity of the left portal branch and inverse increase of the portal vein peak velocity of the right portal branch.

**Figure 7:**
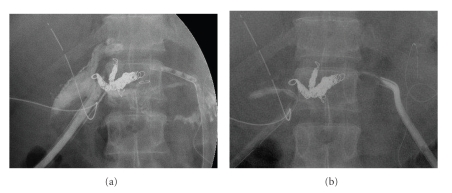
Leakage in the pancreaticojejunostomy (a) is successfully treated by effective drainage (b).

A 55-year-old male underwent pancreaticoduodenectomy as an initial treatment for biliary carcinoma in March, 2009 without preoperative chemoradiotherapy. Histopathological examination revealed papillary adenocarcinoma infiltrating to the fibromuscular layer with no metastasis to the regional lymph nodes, with the pathologic stage of the tumor defined as T1 N0 M0. The drainage tubes were placed at the anterior aspect of the pancreaticojejunostomy and the dorsal space of the hepaticojejunostomy. On the 11th day after surgery, the patient had a spiking temperature of up to 38.5°C with purulent and amylase-rich (>5000IU/l) exudates from the bilateral drainage tubes. The initial clinical impression was a leakage of the pancreaticojejunostomy. Continuous lavage from the drainage tubes using 1.0 L saline /day was begun. Although the fever disappeared after three days, the drainage output of turbid fluids with necrotic tissues persisted. On the morning of the 16th day after surgery, the patient had an episode of hemorrhage from the drainage tubes with a duration of a few minutes, but it spontaneously disappeared with the patient remaining hemodynamically stable. A computed tomography (CT) was performed which demonstrated neither an abscess nor a hematoma around the pancreaticojejunostomy (Figures  1(a) and 1(b)). The stump of the gastroduodenal artery (GDA) showed an outgrowth with a length of 6.0 mm without formation of pseudoaneurysm (Figure 1(b), arrow). The stump of the GDA was immediately adjacent to the tip of the drainage tube placed at the anterior aspect of the pancreaticojejunostomy (Figure 1(b), arrowhead). The sentinel bleeding disappeared just after transfer to the CT room. However, on the evening of the 16th day after surgery, the patient had an episode of back pain, followed by release of 500 mL of hemorrhagic fluid from the drainage tubes. There was no aneurysm or hematoma in the peritoneal cavity visualized with a second CT. The remnant stomach was markedly dilated, being filled with intralumanal fluids. The patient did not vomit, but had severe, burning epigastric pain. An aspiration of the gastric contents yielded 300 mL of hemorrhagic fluids. During the period between the 16th and 17th day postsurgery, the patient had been hemodynamically stable. On the evening of the 17th day postsurgery, the patient vomited large amounts of coffee-grounds liquid with fresh blood in the abdominal drains. Despite the patient being hemodynamically stable, a drop in hemoglobin necessitated transfusion of 2 units of packed RBC. Upper endoscopy revealed a stomach filled with blood but the bleeding point was unable to be identified around the gastrojejunostomy or pancreaticojejunostomy (Figures  1(c) and 1(d)). The patient was kept hemodynamically stable during the 12 hours after the upper endoscopy without bleeding from abdominal drains or hematemesis. On the morning of the 18th day postsurgery, following the appearance of blood in the abdominal drains and hematemesis, copious bloody diarrhea was observed. The resistance of the abdominal wall had increased and a pulsatile mass was palpable at the left upper quadrant. In view of the patient being hemodynamically unstable, a visceral angiogram was performed immediately after fluid resuscitation and blood transfusion of 4 units of packed RBC. The patient underwent emergency angiography using the standard Seldinger technique and an angiography catheter. A selective celiac angiogram showed that the splenic artery and the left gastric artery were all patent with good organ perfusion. A 2.7 cm diameter pseudoaneurysm arising from the stump of the GDA was identified (Figure 2(a), arrow). It is generally accepted that gastroduodenal artery pseudoaneurhysm embolization with stent grafting of the common hepatic artery have significant clinical benefits to maintain liver blood flow [7–9]. However, stent-graft materials adapted to the common hepatic artery could not be prepared for the patient under the emergency conditions. Therefore, coil embolization, both proximal (common hepatic artery) and distal (right and left hepatic artery) of the pseudoaneurysm was performed with microcoils (MWCE-18S-TORNADO) (Figures 2(b) and 2(c). The clinical course and schematic presentation are shown in Figures  3 and 4. Bleeding from the abdominal drains and gastric tube disappeared about 48 hours after coil embolization. The hemoglobin value had decreased to 6.2 g/dL but gradually returned to 8.0 g/dL without blood transfusion. On the 3rd day postcoil embolization, the patient suffered a fever (up to 39°C). Coagulation tests on day 5 postcoil embolization revealed a marked elevation of fibrin degradation product (FDP). CT of the abdomen revealed that segments 2/3 of the liver were replaced by a low density area located near the edge of the liver (Figure 5(a)). The source of high fever was proved to be a hepatic infraction of segments 2/3. Because the right main branch of the portal vein was clearly visualized with a high value of portal vein peakvelocity (PVPV) on a doppler ultrasonography (Figure 6), we believed that severe hepatic failure would not occur. Symptomatic treatment with an initial dose of an antibiotic and vasodilator was started. High fever around 38°C to 39°C persisted for 10 days. Although the high fever resolved spontaneously over the following days, there was a high fever relapse of up to 40°C on the 16th day after coil embolization. CT demonstrated that segments 2/3 of the liver were replaced by fluid-level components with accumulation of gases (Figure 5(b)). We concluded that the hepatic infarction had led to an intrahepatic biloma and abscess formation. High output of purulent fluids from a decompression tube placed intraluminally at the hepaticojejunostomy was observed, suggesting that the formation of the hepatic abscess was associated with a delayed ischemia of the biliary ducts caused by the embolization of the hepatic artery. Conservative treatment with antibiotics was given based on cultures of fluids from the decompression tube and the patient became afebrile on the 30th day postcoil embolization. On the 32nd day after coil embolization, a CT scan revealed that segments 2/3 of the liver were replaced by a homogenous low-density area with thick capsule formation (Figure 5(c)). In addition, anastomotic leakage persisted with a high-output pancreatic fistula from the drain adjacent to the pancreaticojejunostomy (Figure 7(a)). Continuous irrigation from the drain with saline and enteral nutrition from the jejunostomy catheter yielded complete relief of symptoms associated with anastomotic leakage on the 34th day after coil embolization (Figure 7(b)). A solid diet was started on the 40th day after coil embolization. All drains and tubes were removed until the 50th day postcoil embolization. On the 68th day after coil embolization, the patient suffered high fever with epigastric pain. A hepatic abscess was localized by CT in the edge of segments 2/3 penetrating to the lesser sac, which was immediately treated by percutaneous drainage (Figure 5(d)). The patient recovered uneventfully and was discharged on the 101st day after coil embolization. Two months after discharge a CT scan showed disappearance of segments 2/3 of the liver with no abscess formation present (Figure 5(e)). The patient underwent no postoperative adjuvant therapy. The patient is in good condition without any recurrence in the 13 months since recovery.

## 3. Discussion

Urgent laparotomies to control and repair gastroduodenal arterial stumps are reported to be rarely successful, due to the extensive inflammation and necrosis around the pancreatic anastomosis, whereas selective angiography and TAE is now considered the standard therapeutic management with definitive hemostasis achieved in up to 80% of cases [10]. 

On the basis of these descriptions, we believe that appropriate therapy successfully improved the life-threat status in the present case. However, the clinical course included two major problems. First, we should verify whether adequate diagnostic tools could be used to detect postoperative hemorrhages from a pseudoaneurysm of a GDA stump. In the present case, a minor bleed from the surgical drain was observed at 16 days postoperatively, approximately 36 hours before the active life-threatening hemorrhage. This event corresponds to “sentinel bleeding” described by Brodsky and Turnbull [11]. During the periods between sentinel bleeding and embolization under angiography, upper endoscopy and CT were performed twice, with no detection of the origin of bleeding. Limongelli et al. argued that endoscopy is usually used as a first diagnostic tool when an upper gastrointestinal hemorrhage is suspected, but may often fail to identify the site of bleeding, and positive findings such as erosive gastritis on endoscopy can be dangerously misleading and result in a delayed intervention, and in the worst cases, death [1]. Also in the present case, an upper endoscopy on the 17th day postsurgery revealed ulcerative lesions at the gastrojejunostomy and pancreaticojejunostomy. In the present case, the patient had episodes of vomiting of fresh blood, suggesting intestinal bleeding. Schürmann et al. [12] reported that intra-arterial CT mesentericography (CTM) is superior to conventional mesentericography in detection of severe obscure overt intestinal bleeding. To differentiate the bleeding originating from the intestine in postpanceratectomy hemorrhages, CTM can be a useful diagnostic tool. 

On the basis of the following three descriptions, from a review of the literature, we should have performed an emergency angiography when the sentinel bleeding occurred. First, the majority of cases of delayed hemorrhage associated with pseudoaneurysm after PD arise from anastomotic leakage at the pancreaticojejunostomy or a pancreatic fistula [13, 14]. Second, a recent analysis of 1669 consecutive pancreatic resections has demonstrated that angiography is a first diagnostic tool when delayed postpancreatectomy hemorrhage occurs, whether it appears intraluminally or extraluminally [3]. Third, Tien et al. reported that angiography for seven (35%) of 20 patients with sentinel bleeding after PD yielded embolization of pseudoaneurysms before massive bleeding [15]. Tien et al. also revealed that abdominal CT was used as the initial diagnostic tool when sentinel bleeding was detected in 13 patients after PD, but the precise bleeding focus could not be determined in nine of the 13 cases, suggesting that CT is not suitable as a first diagnostic tool when sentinel bleeding occurs. However, in the present case when sentinel bleeding occurred, a CT scan in the arterial phase revealed the long stump of the GDA to be 6.0 mm in diameter. Because the GDA was ligated at its root, it is generally accepted that the stump of the GDA cannot be visualized on CT scans in the arterial phase after PD. We retrospectively speculate that the long stump of the GDA was a predisposing condition for the formation of the poseudoaneurysm or the early stage of pseudoaneurysm.

 Complications associated with hepatic ischemia include liver infarction and bleeding pseudoaneuaneurysms after PD. The incidence of liver abscess after embolization can be more than 30% and transient ischemia and hepatic failure can also occur [6]. During PD, collateral vessels in the hepatoduodenal ligament, which carry the arterial input to the biliary tree, are widely transected. Therefore, it is reasonable to postulate that liver abscesses formed after embolization of the hepatic artery arise from interruption of the arterial flow to the biliary tree. 

Noun et al. reported a case of biliary ischemia following embolization of a pseudoaneurysm after PD, which was treated by percutaneous drainage and corresponded to an intrahepatic biloma, suggesting that the development of this complication can be explained by biliary ischemia rather than parenchymal ischemia [16]. However, to evaluate hepatic complications after embolization of the hepatic artery, more detailed understanding of the clinical course after embolization is required. 

In the present case, we focused on the morphologic change in CT scans during the periods after embolization, and we speculate that the appearance and resolution of the hepatic abscess corresponded to the following five stages: stage 1: on day 3 after embolization, hepatic parenchyma throughout segments 2/3 showed low-density, maintaining the form of a peripheral edge; stage 2: on the 16th day after embolization, edematous changes around the umbilical portion appeared and the intrahepatic Glisson's capsule in segments 2/3 was replaced by accumulation of gases; stage 3: by the 30th day after embolization, the segment 2/3 of the liver was replaced by fluid-level components with capsule formation. Portal branches to the segment 2/3 were not visualized in the portal phase; stage 4: on the 68th day after embolization, the size of the fluid-filled space reduced spontaneously with penetration to the lesser omentum; stage 5: 161 days after embolization, a complete disappearance of the segment 2/3 was observed. It was not possible to evaluate collateral vessels from the emergency angiography. The clinical course after embolization initially (stage 1 and stage 2) reflected a biliary ischemia induced by blocking of the arterial blood supply via the bile ducts. The high-output discharge of purulent fluids from the decompression tube placed at the hepaticojejunostomy, and gas accumulation along with the Glisson's capsule of the segment 2/3 also supported the theory that necrosis of biliary ducts was induced by embolization. However, ischemia of the biliary ducts on its own is insufficient to explain the mechanism for later hepatic events after embolization. In the present case, CT performed on the 30th day postembolization showed complete obstruction of the portal branches to the subsegments (S2 and S3). In addition, we demonstrated by doppler ultrasonography the gradual decrease in portal vein peak velocity (PVPV) of the left main branch and the inverse reaction in the right main branch. Sugimoto et al. reported that the median value of PVPV in cirrhosis was 0.149 m/sec [17]. Therefore, in the present case, we believe that a marked decrease of portal blood flow to the lateral segment also occurred in later stages (stage 3 to 5). Given that the clinical course in the present case led to the spontaneous destruction and disappearance of the segment 2/3, it is reasonable to postulate that a complete defect in the blood supply to the segment 2/3, including the arterial and portal supply, is associated with the observed hepatic complication. Miller and Mineau also inferred that hepatic infarction is likely to be secondary to decreased portal perfusion from hemorrhagic shock coupled with transcatheter occlusion of the hepatic artery [18]. 

 The mechanism for portal obstruction after coil embolization of the hepatic artery remains unclear. We assume that arterial embolization induced edema and inflammation of Glisson's capsule leading to stenosis or obstruction of the portal tract. Miura et al. demonstrated that 4 postpancreatectomy patients, who underwent embolization of the hepatic artery following GDA stump hemorrhages, developed hepatic infarction leading to abscess formation. Interestingly, abscesses were localized in the lateral segment in all four cases [6]. However, it is unclear whether the portal branches of segment 2/3 are more easily obstructed by such conditions. Generally, extrahepatic collateral vessels including the right inferior phrenic artery, renal capsular artery, and intercostals artery, develop early in the bare area of the liver [19]. Accordingly, the right lobe of the liver is predominantly supplied by extrahepatic collateral arteries. Although these collateral vessels were not evaluated on emergency angiography in the present case, such an anatomical background suggests that the right lobe is mainly supplied by collateral arteries after hepatic artery embolization. We speculate that ischemia of the left lobe progressed through this condition where lack of arterial supply occurred after hepatic artery embolization.
